# Psychological interventions for migraine: a systematic review

**DOI:** 10.1007/s00415-016-8126-z

**Published:** 2016-05-09

**Authors:** Andrew Sullivan, Sian Cousins, Leone Ridsdale

**Affiliations:** King’s College London, Denmark Hill Campus, PO57, London, SE5 8AF UK

**Keywords:** Migraine, Headache, Systematic review, Relaxation, Cognitive behavioural therapy, Biofeedback

## Abstract

Migraine causes major health impairment and disability. Psychological interventions offer an addition to pharmacotherapy but they are not currently recommended by the National Institute of Clinical Excellence (NICE) or available in the National Health Service. We aimed to systematically review evidence on the efficacy of psychological interventions for migraine in adults. A search was done of MEDLINE, psychINFO, http://www.opengrey.eu, the meta-register of controlled trials and bibliographies. Twenty-four papers were included and rated independently by two people using the Yates scale, which has 35 points. Cochrane recommendations are that high quality reports score above the mid-point (18 points). Methods used in 17/24 papers were rated ‘high quality’. However, frequently descriptions of key areas such as randomisation methods were omitted. Eighteen studies measured effects of psychological interventions on headache-related outcomes, fifteen reporting significant improvements, ranging 20–67 %. Interventions also produced improvements in psychological outcomes. Few trials measured or reported improvement in disability or quality of life. We conclude that evidence supports the efficacy of psychological interventions in migraine. Over half of the studies were from the USA, which did not provide universal health care at the time of the study, so it is difficult to generalise results to typical populations in receipt of publically funded health services. We agree with the NICE recommendation that high quality pragmatic randomised controlled trials are needed in the UK.

## Introduction

Migraine is a profoundly debilitating condition ranked by the World Health Organisation (WHO) as one of the top 20 causes of disability worldwide [1]. It results in loss of quality of life (QoL) as well as having a significant impact on society as a whole. In the United Kingdom (UK), approximately 25 million work days are lost to migraine each year, with headache disorders estimated to cost the economy in excess of £5 billion per year [2, 3]. Current treatment for migraine is primarily focussed on pharmacological interventions, however, these treatments only show moderate efficacy. With headache disorders now considered a bio-psychosocial phenomenon, pharmacotherapy fails to address underlying psychological and social factors influencing headache [4]. Evidence also shows that migraine may be comorbid with psychiatric conditions, notably anxiety and depression [5]. As such, psychological interventions are considered as a possible alternative or adjunct to pharmacotherapy.

The main psychological interventions employed as treatment for migraine include relaxation training (RT), cognitive behavioural therapy (CBT) and biofeedback (BF). Despite over 40 years of research into these treatments and endorsement by organisations worldwide including the US Headache Consortium and WHO, they are not currently recommended for use in migraine patients in the UK [6, 7]. However, in 2012, the National Institute of Clinical Excellence (NICE) issued a research recommendation for a pragmatic randomised controlled trial (RCT) to be conducted to determine the efficacy of psychological interventions for treatment of chronic headache, perhaps paving the way for future provision of these interventions in UK clinical practice [8]. To this end, a pilot trial was undertaken at King’s College London to assess the feasibility of trialling CBT combined with RT for chronic migraine in adults [9]. In this context, we reviewed the literature on psychological interventions for migraine at this time.

Goslin et al. [10] previously systematically reviewed psychological interventions for migraine in 1999 concluding that BF, RT and CBT have modest efficacy. Subsequent systematic reviews have since focussed on BF and paediatric populations [11–13]. Therefore, an up-to-date overview of the psychological interventions for adult migraineurs is currently needed. In light of this, we aimed to systematically review the evidence regarding the efficacy of psychological interventions for treatment of adult migraine since 1999.

## Methods

### Selection criteria

Trials were included if they (1) included participants with a diagnosis of migraine; (2) employed BF, RT and/or CBT as an intervention; (3) were published from 1999 to 2014; (4) were a RCT; (5) were in English.

Studies were excluded if (1) they did not report a specific headache diagnosis; (2) they included populations of other headache disorders such as cluster headache; (3) they employed non-psychological interventions such as physical therapy; (4) there were no results published; (5) only physiological outcomes were reported.

Studies with mixed populations of migraine and tension type headache (TTH) were included because, these disorders represent a heterogeneous group and to exclude such studies would exclude a significant part of the migraine literature. Goslin et al. [10] also included such studies in their review.

### Search strategy

An electronic search was carried out, for published and unpublished trials, of the databases (1) MEDLINE; (2) psychINFO; (3) opengrey.eu; and (4) the meta-register of controlled trials. This was carried out using the key words “migraine disorder”, “migraine with aura”, “migraine without aura”, “migraine” and “migraine headache” combined with “cognitive therapy”, “behaviour therapy”, “cognitive behavioural therapy”, “relaxation therapy”, “relaxation training” and “biofeedback”. A manual search of relevant bibliographies was also performed.

### Quality assessment

Texts included were quality assessed by two independent people using the Yates scale [14]. After one round of ratings, they were compared, and if there were disagreements, raters reassessed in a second round of ratings. Subsequently, further disagreements were taken to the principal investigator (Leone Ridsdale) for resolution. The Yates scale is scored out of 35 points with 26 items assessed, including some specific to psychological interventions such as assessment of therapist training and treatment expectations. This scale has been deemed to have good construct validity and reliability [15]. Furthermore, it has had a rigorous development through a standardised procedure [14, 15]. The ‘therapist training’ criterion of the scale was excluded when a therapist was irrelevant to the intervention such as in ‘self-help’ treatments. In this case, trials were scored out of 33 instead of 35. A percentage was calculated from the final score so that trials could be compared regardless of whether they were scored out of 33 or 35. A Cochrane review used the mid-point (score of 18) as the divider between a ‘high quality’ and ‘low quality’ study [16]. So with ratings converted to percentages in this review, a score ≥50 % was deemed high quality and a score ≤49 % was deemed low quality.

## Results

**Fig. 1:**
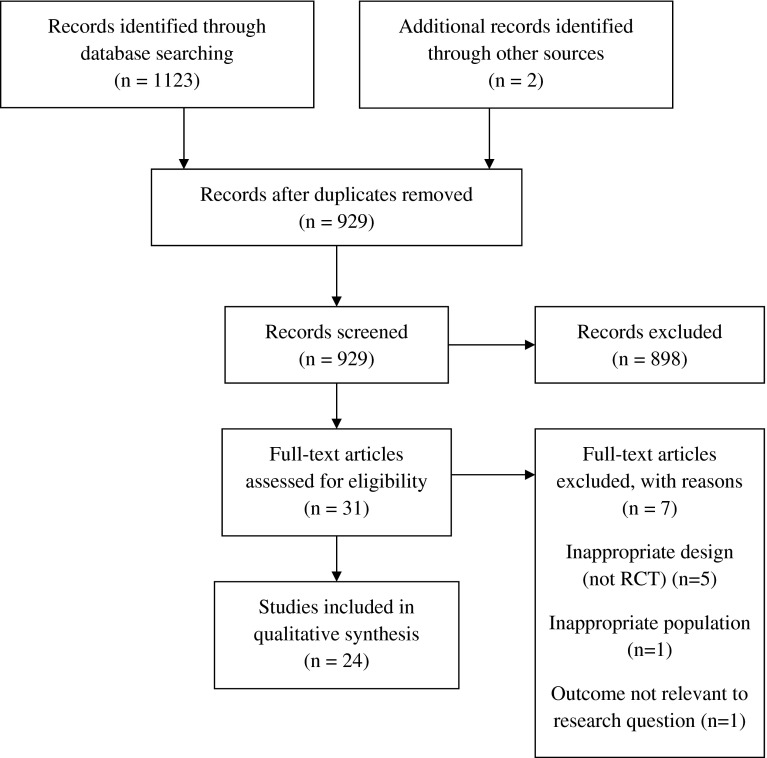
PRISMA flow diagram

The initial database search returned 1123 hits with a further two records identified through bibliographic searching. Following screening and full text assessment, 24 publications were included in the review. Figure 1 shows the PRISMA flow diagram of the review process.

Table 1 shows a summary of publications included in the review. Taking account of secondary analyses (see Table 1: 3b, 12b, 17b and 17c) and follow up studies (see Table 1: 10b), there were 19 separate studies.

**Table 1:** Study characteristics, quality and effect on daily self-report headache frequency

#	Study	Diagnosis (sample size)	Intervention	Duration	Control	Outcome measures	Quality	Percentage change in daily self-report headache frequency from baseline to endpoint, when reported (duration of follow up)
*CBT*								
1	Thorn et al. [17]	Migraine/TTH (34)	Group CBT	10 weeks (10× 1.5 h sessions)	Wait-list	Headache frequency, intensity (diary); psychological variables (BDI, BAI, PCS, PASS, HMSE)	22/35 (63 %)	−8 % non-significant (none: post-intervention only)
*Relaxation therapy*								
2	Devineni and Blanchard [18]	Migraine/TTH (156)	Internet based relaxation training	6 weeks	Wait-list	Headache index (diary), HSQ; psychological variables (CES-D, STAI); Disability (HDI)	17/33 (52 %)	N/A^a^
3a	D’Souza et al. [19]	Migraine/TTH (140)	Written emotional disclosure or audiotape relaxation training	2 weeks (4 sessions)	Unemotional writing	Headache frequency (diary + retrospective) severity (retrospective); Disability (MIDAS); SCL-90R	18/33 (55 %)	Relaxation: −42 % Written emotional disclosure: +19 % non-significant (3 months)
3b	Kraft et al. [20]	Migraine (90)	As above	As above	As above	Headache frequency, MPQ; psychological measures (EAC, HMSE, PANAS); Disability (HDI)	12/33 (36 %)	N/A^b^
4	Dittrich et al. [21]	Migraine (30)	Exercise + relaxation	6 weeks (12× 1 h sessions)	Information giving	Headache frequency, intensity (retrospective); Psychological variables (BDI, FKB-20), QoL (PLC)	11/35 (31 %)	N/A^c^
5	Varkey et al. [22]	Migraine (91)	Physio led relaxation training	3 months (6 sessions)	Exercise or topiramate	Headache frequency, days, intensity, medication (diary); QoL (MsQoL)	26/35 (74 %)	−23 % (6 months)
*Biofeedback*								
6	Vasudeva et al. [23]	Migraine (40)	Biofeedback-assisted relaxation	12 weeks (12× 50 min sessions)	Self-relaxation	Cerebral blood flow velocity, headache index (log sheets), psychological variables (BDI, STAI-S)	14/35 (40 %)	N/A^a^
7	Kang et al. [24]	Migraine (32)	Biofeedback-assisted autogenic training	4 weeks (8× 50 min sessions)	Simple biofeedback	Headache index (diary), MPQ, CGI-S; psychological variables (HAM-A, HAM-D, STAI-S)	15/35 (43 %)	N/A^a^
*CBT* + *relaxation therapy*								
8	Lemstra et al. [25]	Migraine (84)	Exercise, stress management lecture, relaxation therapy lecture, massage therapy	6 weeks	Wait-list	Headache frequency, intensity, medication consumption (retrospective); psychological variables (BDI); disability (PDI); QoL (visual analogue scale)	22/35 (63 %)	N/A^c^
9	Andersson et al. [26]	Migraine/TTH (44)	Internet based self-help + therapist phone call	6 weeks (6× modules + 6× 20 min phone call)	Internet based self-help only	Headache index, frequency, intensity (diary); psychological variables (HADS, CSQ, PSS); disability (HDI)	17/33 (52 %)	+1 % non-significant (none: post-intervention only)
10a	Mérelle et al. [27]	Migraine (129)	Group based behavioural training delivered by lay trainers	10 weeks (7× 2 h sessions)	Wait-list	Headache frequency, intensity (diary); psychological variables (HSLC, HMSE); QoL (MSQoL); disability (MIDAS); SF-36	25/35 (71 %)	−21 % (none: post-intervention only)
10b	Mérelle et al. [28]	Migraine (129)	As above	As above	As above	As above	26/35 (74 %)	−29 % (6 months)
11	Fritsche et al. [29]	Migraine (182)	Bibliotherapy + minimal-contact behavioural therapy delivered by psychotherapists	5 weeks (5× 2 h sessions)	Bibliotherapy	Medication intake days, headache days, intensity (diary); psychological variables (HADS, CPAQ, KKG, PRSS); disability (diary)	23/35 (66 %)	−24 % (12–24 months)
12a	Hedborg and Muhr [30]	Migraine (83)	Internet based MBT	6 months	Self-relaxation	Headache days (diary); psychological variables (MADRS-S); QoL (PQ23)	18/33 (55 %)	(Significant decrease but no raw data provided)
12b	Hedborg and Muhr [31]	Migraine (83)	As above	As above	As above	Medication consumption, efficacy (diary)	16/33 (48 %)	N/A^b^
13	Bromberg et al. [32]	Migraine (213)	Internet based ‘pain ACTION’	4 weeks (8× 20 min sessions)	Treatment as usual	Psychological variables (CPCI-42, HSES, PCS, HSLC, DASS-21); disability (MIDAS); PGIC	25/33 (76 %)	N/A^a^
*Biofeedback* + *relaxation therapy*								
14	Kaushik et al. [33]	Migraine (192)	Biofeedback + home based relaxation therapy	6 months (10× sessions of biofeedback)	Propranolol	Headache resurgence rate, frequency, intensity, index (daily self-report); physiological variables; well-being (visual analogue scale)	20/35 (57 %)	−52 % (6 months)
15	Mullally et al. [34]	Migraine/TTH (64)	Biofeedback + relaxation therapy delivered by a pain clinician	6 weeks (10× 50 min sessions of biofeedback)	Relaxation alone	Headache frequency (retrospective) medication consumption	12/35 (34 %)	N/A^c^
*CBT* + *relaxation therapy* + *biofeedback*								
16	Martin et al. [35]	Migraine/TTH (64)	CBT + relaxation training delivered by psychologists, or temporal pulse biofeedback	8 weeks (8× 1 h sessions)	Wait-list	Headache ‘rating’ (daily cards); psychological variables (CAI, CSI, HSES, HSLC)	20/35 (57 %)	N/A^a^
17a	Holroyd et al. [36]	Migraine (232)	Beta blocker/placebo + behavioural management (workbook, audio lessons, migraine management sessions delivered by psychologists, home based biofeedback)	4 months (4× 1 h sessions of migraine management, 10× audio lessons)	Beta blocker/placebo alone	Headache frequency, characteristics, medication use (diary); QoL (MSQoL)	27/35 (77 %)	Behavioural management + Beta-blocker: −67 % (12 months) Behavioural management + placebo: −48 % (12 months)
17b	Seng and Holroyd [37]	Migraine (176)	As above	As above	As above	Psychological variables (HMSE, HSLC)	27/35 (77 %)	N/A^b^
17c	Seng and Holroyd [38]	Migraine (177)	As above	As above	As above	Headache frequency, characteristics (diary); Disability (HDI); QoL (MSQoL)	25/35 (71 %)	N/A^b^
*Miscellaneous*								
18	Calhoun and Ford [39]	Migraine (43)	Behavioural sleep instructions	N/A	Placebo instruction	Headache frequency, index (diary)	13/35 (37 %)	−28 % (12 weeks)
19	Wachholtz and Pargament [40]	Migraine/TTH (92)	Meditation	2 weeks (20 min/day)	Muscle relaxation	Headache days, severity (diary); Psychological measures (PANAS, STAI, HMSE); QoL (MSQoL); spiritual measures	21/35 (60 %)	−37 % (none: post-intervention only)

*BAI* beck anxiety inventory, *BDI* beck depression inventory, *CAI* cognitive appraisal inventory, *CES-D* Centre for Epidemiologic Studies Depression Scale; *CGI-S* Clinical Global Impression severity scale; *CPAQ* Chronic Pain Acceptance Questionnaire; *CPCI-42* Chronic Pain Coping Inventory-42, *CSI* coping strategies inventory, *CSQ* coping strategies questionnaire, *DASS-21* Depression Anxiety Stress Scales, *EAC* Emotional Approach Coping Scale, *FKB-20* Fragebogen zum Körperbild (German), *HADS* Hospital Anxiety and Depression Scale, *HAM-A* Hamilton Rating Scale for Anxiety, *HAM-D* Hamilton Rating Scale for Depression, *HDI* headache disability inventory, *HSES* Headache Self-efficacy Scale, *HSLC* headache specific locus of control, *HSME* Headache Management Self-efficacy Scale, *HSQ* headache symptom questionnaire, *KKG* questionnaire for assessment of control beliefs about illness and health, *MADRS*-S-Montgomery-Asberg Depression Rating Scale; *MIDAS* migraine disability assessment questionnaire, *MPQ* McGill pain questionnaire, *MSQoL* migraine specific quality of life, *PANAS* positive and negative affect schedule, *PASS* Pain Anxiety Symptom Scale, *PCS* Pain Catastrophizing Scale, *PDI* Pain Disability Index; *PGIC* patient global impression of change, *PLC* Profil der Lebensqualität chronisch (German), *PQ23* quality of life questionnaire, *PRSS* Pain-related Self-Statements Scale, *PSS* Perceived Stress Scale, *SCL-90R* symptom checklist-90-R, *SF-36* Short Form 36 Health Survey, *STAI* state-trait anxiety inventory

^a^Did not measure headache frequency

^b^Secondary analysis

^c^Retrospective headache frequency measurements used

Twelve studies included a population with a diagnosis of migraine only, and seven included populations with a diagnosis of migraine and/or TTH. Ten studies were based on North American populations; the remainder were European (*n* = 6), Asian (*n* = 2) and Australian (*n* = 1), with none from the UK.

Trialists tended to opt for interventions consisting of a combination of psychological treatments, with CBT + RT, the most commonly adopted approach (*n* = 6). Other treatment combinations included BF + RT (*n* = 2) and combinations of all three modalities (*n* = 2). One study employed CBT on its own, four RT on its own and two BF on its own. Two studies used interventions that did not strictly fall into the CBT, RT or BF category. These employed meditation and behavioural sleep management as interventions, which were deemed directly related and so were included in the review.

Comparison groups used in the studies were variable (see Table 1 for details). Most commonly, a wait-list control group was used (*n* = 5). Other control groups used included pharmacological interventions, self-help and self-relaxation.

The outcome measures used fell into four categories: headache, psychological, disability and QoL. Eighteen studies directly measured the effect of the intervention on headache outcome measures. Fifteen of these reported that psychological interventions significantly improved headache outcome measures ranging from 20 to 67 %. Eleven studies reported headache frequency/days as measured by daily self-reporting, the recommended outcome measure for headache trials [41, 42]. Studies showed a 21–67 % improvement in this measure, after intervention (see Table 1). The largest improvement was seen when a combination of CBT, RT and BF were provided in conjunction with pharmacotherapy [36]. Fifteen studies assessed psychological outcomes with four out of eight studies reporting significant improvements in anxiety and six out of ten reporting improvements in depression, ranging 14–32 % and 18–62 %, respectively. Eight studies assessed disability with psychological interventions yielding improvements of 28–44 % in four of the studies. Seven assessed impact on QoL, with three reporting improved QoL following intervention, ranging from 5 to 39 %.

Seventeen out of the 24 publications were graded as high quality. Nevertheless, descriptions of key areas of methodology were omitted. For example, despite all publications reporting that participants were randomised, only nine provided an adequate description of randomisation. Similarly, publications often failed to report how they minimised allocation bias and measurement bias. Only four publications were deemed to have adequate control groups that were well matched to the intervention group and only three used outcome measures that were validated. Only one study was blinded to study participants, but this is difficult in complex-intervention trials, and only one study assessed the treatment expectations of study participants.

## Discussion

The range of efficacy of psychological interventions was broad, from 20 to 67 %. There was no evidence to indicate that one approach of CBT, RT or BF was superior to another. Since Goslin et al. [8] last reviewed the literature in 1999, the most favoured behavioural approach to migraine has been CBT + RT, in particular, minimal-contact interventions. These low intensity interventions demonstrated a modest efficacy in migraine reduction, which is of particular relevance because, such approaches are likely to be less costly, hence, potentially more cost-effective [43]. A recent paper provides some understanding of patients’ views using qualitative methods, with interviews [9, 44]. Combining trial methodology with qualitative methods is recommended by the Medical Research Council, but so far not used in trials of psychological interventions for migraine [45]. The study of minimal-contact CBT + RT reported that participants found the relaxation aspects of therapy easier to implement. CBT components of therapy were more challenging to learn and apply in the context of a minimal-contact intervention [44]. Improvements in headache seen in this review in studies using CBT related interventions are less than those reported by Goslin et al. [10]. This is in part due to more intensive approaches used by earlier studies. Higher contact therapy, unsurprisingly, has had a larger effect than minimal contact, so a balance must be struck to maximise efficacy and minimise cost [46].

In our review, we note a large range in the efficacy of psychological interventions for migraine. Differences in the intensity of therapeutic contact may in part explain this. However, it may also be attributed to diversity in therapeutic interventions that make up CBT, RT and BF. For example, of the studies that employed RT as part of their intervention, both autogenic training and progressive muscle relaxation were employed in different studies as well as combinations of the two. CBT interventions were particularly diverse, combining various aspects of education and management strategies for triggers, stress and fear among others. This significant heterogeneity within intervention types makes it difficult to compare results and ascertain if there is an optimum therapy design. Few studies compare the effect of behavioural interventions with pharmacological interventions; however, of the two that did, no significant differences in efficacy were noted [22, 33]. Pharmacotherapy and behavioural therapy may be complementary in nature with the greatest magnitude of reduction (67 %) in headache frequency achieved by implementing a combination of the two [36].

Studies in our sample were often lacking in quality in key areas. Problems in methodology were similarly reported in the review by Rains et al. [47]. In our sample, poor reporting of randomisation methods was common. Blinding was also a challenge, with only 1 study blinding subjects. However, considering that blinding in psychological interventions is often not possible, one could assess the expectations of patient to treatment as an alternative. Still, only 1 reported such an assessment, making excluding ‘placebo’ effects difficult. Few studies used outcome measures that were all considered valid. This is in part because, the recommended outcome measure for headache trials is daily self-report headache frequency/days, which strictly speaking is not a validated measure, therefore, the Yates scale may have shown unnecessary bias against these studies [41, 42].

There were several limitations to our review. Firstly, studies included populations of not only migraine but also TTH. This was done to ensure that we included as much of the migraine trial evidence as possible. However, we cannot be sure with these studies whether treatment effects were due to effects on migraine or TTH or both; this may also be a reason behind the wide range in efficacy that interventions appeared to have. Secondly, the outcome measures of the studies in our sample were heterogeneous. This makes it difficult for us to make a comparison between all of the studies and draw solid conclusions regarding efficacy. We have illustrated percentage reduction of headache frequency as measured by prospectively recorded self-report measures in Table 1. This is a recommended outcome measure, however, the number of studies using this was limited. We did not include other headache outcome measures such as headache index because, these are not favoured by guidelines [41]. Furthermore, headache frequency reported retrospectively was not included because, they are less reliable than prospective studies of headache frequency [41, 48]. In future, the use of a core outcome set would reduce heterogeneity and strengthen the evidence-base for psychological interventions for migraine.

The evidence included suggests that psychological interventions can be effective for migraine; a significant portion of this evidence favoured a CBT + RT approach. The evidence-base is still lacking in quality, and participants were not generally representative of those receiving publicly universal care, as provided in the National Health Service. The NICE guidelines call for pragmatic RCT’s of psychological interventions for headache [8]. Our pilot trial begins to address this issue and may provide foundations for further testing of psychological interventions for migraine in the UK [9].
